# The Impact of Maternal Metabolic Syndrome on Gross Placental Structure in Term Pregnancies: A Comparative Study

**DOI:** 10.7759/cureus.100869

**Published:** 2026-01-05

**Authors:** Mukund Vatsa, Rashmi Malhotra, Mukesh Singla, Om Kumari, Shailza Yadav, Bharti Jakhar, K. Ankush Patil

**Affiliations:** 1 Anatomy, All India Institute of Medical Sciences, Rishikesh, Rishikesh, IND; 2 Obstetrics and Gynaecology, All India Institute of Medical Sciences, Rishikesh, Rishikesh, IND; 3 Physiology, All India Institute of Medical Sciences, Rishikesh, Rishikesh, IND

**Keywords:** cotyledons, dyslipidaemia, metabolic syndrome, placenta, placental morphometry, umbilical cord

## Abstract

Background and objective

The placenta is a key indicator of the intrauterine environment and directly affects fetal growth. Maternal metabolic syndrome (MetS), which is increasingly common during pregnancy, can impair placental development through chronic inflammation, insulin resistance, and vascular dysfunction. This study aimed to compare the gross morphology of the placenta and umbilical cord in pregnancies complicated by MetS with those in pregnancies without the condition, and to assess associated neonatal birthweight outcomes.

Methods

This cross-sectional comparative study included 60 term placentae, comprising 30 from MetS pregnancies and 30 from control pregnancies. Gross placental parameters assessed included placental weight, length, breadth, thickness, cotyledon number, and umbilical cord length and diameter. Neonatal outcomes assessed included birth weight and the placental to birth weight ratio. Statistical analysis was performed using the Student’s t-test, with a p-value < 0.05 considered significant.

Results

Placentae from MetS pregnancies were significantly smaller and lighter than those from the control group (440 ± 52 g vs. 515 ± 48 g; p < 0.001), with significant reductions in placental length, breadth, and cotyledon number (p < 0.001). Umbilical cord length was also significantly shorter in the MetS group (38.5 ± 5.8 cm vs. 46.4 ± 6.1 cm; p < 0.001). Neonates born to MetS mothers had a lower birth weight (2.65 ± 0.34 kg vs. 3.05 ± 0.28 kg; p < 0.001) and a lower placenta-birth weight ratio (0.162 ± 0.02 vs. 0.171 ± 0.03; p = 0.045), indicating reduced placental efficiency.

Conclusions

Maternal MetS is associated with restricted placental development, reduced placental efficiency, and significant impairment of fetal growth. Routine gross placental morphometry, when paired with neonatal anthropometry, offers a simple, effective tool for identifying fetoplacental insufficiency in MetS pregnancies and underscores the importance of early metabolic risk assessment and improved fetal surveillance.

## Introduction

The placenta is one of the most essential organs of pregnancy, serving as the primary interface between the maternal and fetal circulations. In addition to facilitating the transport of oxygen, nutrients, and waste products, it performs critical endocrine, immunological, and metabolic functions that are necessary for maintaining a healthy intrauterine environment [1,2]. Any disturbance in maternal physiology can directly influence placental growth and structure, and such alterations often present as measurable gross and microscopic morphological changes that may compromise placental efficiency and fetal development [3].

Maternal metabolic syndrome (MetS) has emerged as a major public health challenge worldwide and is increasingly prevalent among women of reproductive age, particularly in India due to rapid urbanization, sedentary lifestyles, dietary transitions, and rising obesity rates [4,5]. MetS comprises a constellation of metabolic abnormalities, including central obesity, hypertension, dyslipidemia, and impaired glucose regulation, which together significantly increase the risk of cardiovascular disease and type 2 diabetes mellitus [6,7]. During pregnancy, this cluster of metabolic disturbances creates a chronic pro-inflammatory, insulin-resistant, and vasoconstricted intrauterine environment [8]. These pathophysiological changes can adversely affect placental vasculogenesis, trophoblastic invasion, uteroplacental perfusion, and nutrient transport, thereby impairing fetal growth [9].

Several studies have independently documented placental alterations in specific metabolic disorders such as diabetes mellitus, chronic hypertension, preeclampsia, and gestational diabetes [10-12]. However, these conditions frequently coexist in women with MetS, resulting in a combined metabolic burden that may exert a greater effect on placental development and function [13]. Despite this, relatively few studies have evaluated placental morphology in pregnancies complicated specifically by MetS as a unified clinical entity [14]. In contrast, gross placental morphometric parameters remain comparatively understudied, despite being among the earliest and most clinically meaningful indicators of placental insufficiency [14,15]. Most published research in this area has focused primarily on microscopic placental alterations, including villous immaturity, syncytial knot formation, fibrinoid necrosis, and vascular wall thickening [15,16]. 

Simple gross measurements such as placental weight, surface dimensions, thickness, cotyledon number, and umbilical cord characteristics reflect the extent of villous arborization, chorionic plate expansion, and overall functional maturity of the placenta, and have been demonstrated to correlate closely with neonatal birth weight and perinatal outcomes [14,15,16,17]. Given the rising prevalence of MetS among Indian women and the limited availability of region-specific morphometric data, the present study was undertaken to assess gross placental structural changes in mothers with MetS delivering at a tertiary care center in Rishikesh, Uttarakhand, in northern India. A clearer understanding of these alterations may facilitate early identification of fetoplacental compromise and assist clinicians in improving antenatal surveillance and perinatal management [18,19,20].

## Materials and methods

Study design and setting

This was a hospital-based, observational, cross-sectional comparative study conducted in the Department of Anatomy in collaboration with the Department of Obstetrics and Gynaecology at the All India Institute of Medical Sciences (AIIMS), Rishikesh, Uttarakhand, India. The study period extended from 17 January 2024 to 4 July 2025. The study was designed and reported in accordance with the STROBE (Strengthening the Reporting of Observational Studies in Epidemiology) guidelines for observational studies [21].

Study population and sample size

A total of 60 placentae obtained from term deliveries were included in the study, comprising 30 placentae from pregnancies complicated by maternal metabolic syndrome (MetS group) and 30 placentae from age and gestational age-matched healthy pregnancies (control group). The sample size was determined based on feasibility and comparison with previously published placental morphometric studies evaluating gross placental parameters, which have commonly included 20-40 placentae per group and demonstrated statistically significant differences in placental dimensions and weight [12]. The mean maternal age was 29.8 ±4.2 years in the MetS group and 30.3 ± 4.5 years in the control group, with no statistically significant difference between the two groups.

Inclusion and exclusion criteria

Placentae from women delivering at term gestation (≥37 weeks) were included. Women in the MetS group were diagnosed during pregnancy based on predefined criteria (described below). The control group included healthy pregnant women without MetS or other major medical comorbidities. Placentae from pregnancies complicated by multiple gestation, congenital fetal anomalies, placental abruption, placenta previa, intrauterine fetal demise, or severe maternal infections were excluded from the study.

Diagnostic criteria for metabolic syndrome

Maternal MetS was diagnosed based on the presence of three or more of the following components, adapted from internationally accepted definitions for metabolic syndrome and routinely used obstetric diagnostic criteria: impaired glucose regulation (gestational diabetes mellitus or pregestational diabetes mellitus); hypertension (chronic hypertension or gestational hypertension); dyslipidaemia, documented antenatally; and central obesity or increased BMI, documented in antenatal records

All women included in the MetS group were receiving standard antenatal care and appropriate medical management for their metabolic conditions as per institutional protocols. Stratification based on degree of metabolic control was not performed and is acknowledged as a limitation. The diagnostic components were adapted from internationally accepted definitions of metabolic syndrome as proposed by Alberti et al. and routinely applied in obstetric and metabolic research [9].

Ethical considerations

The study was approved by the Institutional Ethics Committee of AIIMS, Rishikesh (approval no. AIIMS/IEC/25/447; date: 02/05/2025). Written informed consent was obtained from all participants before their inclusion in the study.

Placental collection and preparation

Immediately after delivery, placentae were collected along with the attached umbilical cord. Placentae were gently washed under running tap water to remove adherent blood clots. The membranes were trimmed, and excess blood was removed. All measurements were performed on fresh specimens to avoid fixation-related shrinkage. The umbilical cord available for measurement represented the placental segment of the umbilical cord, extending from the placental insertion to the cut end, as a portion of the cord remains attached to the neonate at delivery; this approach was applied uniformly across all cases.

Gross placental morphometry

**Figure 1:**
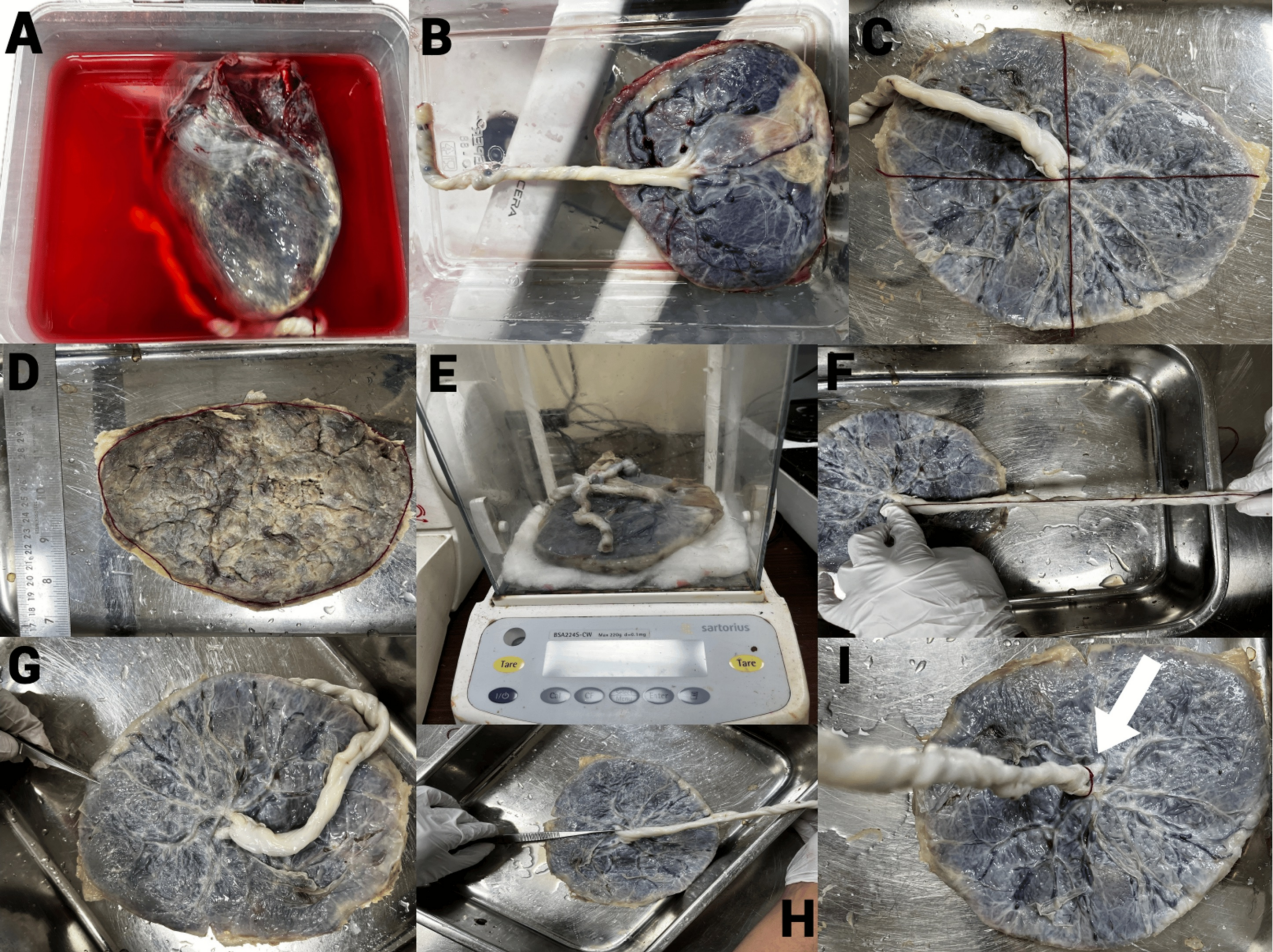
Gross morphological examination of placenta in maternal metabolic syndrome Composite photographic panel showing sequential steps in the gross examination of a freshly delivered placenta from a mother with metabolic syndrome. All measurements were recorded on fresh specimens to avoid fixation-related shrinkage (A) Fresh placenta immersed in normal saline (0.9%) immediately after collection to remove surface blood clots. (B) Fetoplacental unit showing the fetal surface of the placenta with umbilical cord attachment. (C) Maternal surface of the placenta divided into quadrants for systematic cotyledon evaluation. (D) Trimmed placenta with membranes removed, placed on a calibrated flat surface for morphometric assessment. (E) Measurement of fresh placental weight using a calibrated electronic digital weighing scale. (F) Measurement of placental length (maximum diameter) using a non-elastic measuring thread, subsequently measured against a ruler. (G) Measurement of placental thickness at the periphery of the placental disc. (H) Measurement of placental thickness at the centre of the placental disc, aligned in the same orientation as panel (G). (I) Measurement of umbilical cord diameter at the site of placental insertion using vernier callipers

Gross placental examination and morphometric measurements were performed using a standardized protocol, as illustrated in Figure 1. The protocol was originally developed by the authors, based on widely accepted anatomical and obstetric practices routinely employed in placental gross examination and described in placental pathology literature [10,14]. All placentae were examined on fresh specimens following uniform washing and trimming of membranes to remove adherent blood clots. Measurements were obtained using the same calibrated instruments for all cases to ensure standardization.

Placental weight was recorded using a calibrated electronic weighing scale. Placental length and breadth were measured as the maximum diameter and the perpendicular diameter of the placental disc using a non-elastic measuring tape or thread. Placental breadth represents a surface dimension of the placental disc, measured as the diameter perpendicular to placental length. Placental thickness was measured at both the centre and periphery of the placental disc using a measuring scale; however, only the central placental thickness was used for statistical analysis. This approach was adopted because central placental thickness is less affected by marginal tapering and provides a more consistent and reproducible gross thickness measurement across specimens. Cotyledon number was determined by visual inspection of the maternal surface after dividing it into quadrants to ensure systematic and reproducible counting.

Placental shape classification

Placental shape was classified into predefined morphological categories and coded numerically for statistical analysis, as described in placental morphometric and surface shape studies [14,15]. Placental shape was treated as a categorical variable and numerically coded as follows: Code 1 - Discoid: Nearly circular placenta with smooth, uniform margins, representing the normal placental morphology. Code 2 - Oval: Elliptical placenta with one axis longer than the other. Code 3 - Irregular: Placenta with uneven, indented, or lobulated margins not conforming to circular or oval outlines. Code 4 - Bilobed/Succenturiate: Placenta consisting of two nearly equal lobes or the presence of an accessory lobe.

Each placenta was independently evaluated by two anatomists. In cases of discrepancy, reassessment was performed jointly until consensus was achieved. The numeric codes were used solely to facilitate statistical comparison and do not represent continuous variables. As placental shape is a categorical morphological parameter, the data were expressed as median (interquartile range (IQR).

Umbilical cord measurements

Umbilical cord length was measured from the placental insertion point to the cut end of the cord using a non-elastic measuring tape or measuring thread, subsequently measured against a ruler. Umbilical cord diameter was measured at the site of placental insertion using vernier callipers. The number of umbilical vessels was assessed grossly at the cut end of the umbilical cord. All placentae demonstrated the normal configuration of two umbilical arteries and one umbilical vein. Measurement of individual arterial and venous diameters was not performed, as the study focused on gross umbilical cord morphometry, including overall cord length and external diameter.

Umbilical cord length was measured from the placental insertion point to the cut end of the cord using a non-elastic measuring tape or thread and ruler. Umbilical cord diameter was measured at the site of placental insertion using vernier callipers and/or thread and ruler, where appropriate. The number of umbilical vessels was assessed grossly; all placentae demonstrated the normal configuration of two umbilical arteries and one umbilical vein.

Maternal and neonatal data

Maternal demographic and obstetric variables, including maternal age, gestational age at delivery, parity, and mode of delivery, were recorded. Neonatal outcomes included birth weight and placenta-birth weight ratio. Maternal obstetric variables, including parity, were recorded and compared between the MetS and control groups. Mode of delivery (vaginal or caesarean section) was recorded for all participants and compared between the MetS and control groups.

Statistical analysis

Data were entered into Microsoft Excel and analyzed using SPSS Statistics version 26.0 (IBM Corp., Armonk, NY). Normality of continuous variables was assessed using the Shapiro-Wilk test. Normally distributed variables were expressed as mean ± standard deviation (SD) and compared using the independent samples t-test. Non-normally distributed variables were expressed as median (IQR) and compared using the Mann-Whitney U test. Categorical variables were analyzed using the Chi-square test or Fisher’s exact test, as appropriate. A p-value < 0.05 was considered statistically significant.

## Results

The standardized steps involved in gross placental examination and morphometric assessment, as described in the Materials and Methods section, are summarized in Figure 1.

Demographic characteristics

The demographic characteristics of participants in the MetS and control groups are presented in Table 1. There was no statistically significant difference between the two groups in terms of maternal age, gestational age at delivery, parity, or mode of delivery (p > 0.05 for all), suggesting comparability between the groups. The MetS group included 30 participants, while the control group also included 30 participants.

**Table 1:** Demographic and baseline maternal characteristics of the study population Statistical comparisons were performed using the Independent samples t-test or Mann-Whitney U test for continuous variables and the Chi-square or Fisher’s exact test for categorical variables. A p-value < 0.05 was considered statistically significant MetS: metabolic syndrome; SD: standard deviation; IQR: interquartile range

Variable	MetS group (n = 30)	Control group (n = 30)	P-value
Maternal age, years, mean ± SD	29.8 ± 4.2	30.3 ± 4.5	0.64
Gestational age at delivery, weeks, mean ± SD	38.2 ± 1.1	38.4 ± 1.0	0.48
Parity, median (IQR)	2 (1–2)	2 (1–3)	0.57
Mode of delivery (vaginal/cesarean), n (%)	18 (60%)/12 (40%)	20 (66.7%)/10 (33.3%)	0.59

Gross placental and umbilical cord parameters

A comparison of gross placental and umbilical cord parameters between the MetS and control groups is presented in Table 2. Based on the predefined placental shape classification, discoid placental shape (code 1) was the most common morphology observed in both the metabolic syndrome and control groups; no statistically significant difference in placental shape distribution was noted between the groups.

**Table 2:** Comparison of gross placental and umbilical cord parameters between MetS and control groups ^†^Placental shape types represent a categorical morphological variable; therefore, no physical unit applies. Shape categories were numerically coded for statistical analysis and presented as median (IQR) The MetS group comprised 30 participants, and the control group comprised 30 participants. Statistical analysis was performed using the Independent samples t-test for normally distributed continuous variables and the Mann–Whitney U test for non-normally distributed variables. A p-value < 0.05 was considered statistically significant MetS: metabolic syndrome; SD: standard deviation; IQR: interquartile range

Parameter	MetS group (n = 30)	Control group (n = 30)	Test statistic	P-value	Interpretation
Placental weight, g, mean ± SD	440 ± 52	515 ± 48	t = 5.46	< 0.001	Significantly reduced placental mass in MetS, suggesting placental insufficiency
Placental length, cm, mean ± SD	17.4 ± 1.5	19.2 ± 1.6	t = 4.14	0.0002	Reduced placental surface area in MetS
Placental breadth, cm, mean ± SD	15.9 ± 1.3	17.5 ± 1.4	t = 4.26	0.02	Decreased area available for maternal–fetal exchange
Central placental thickness, cm, mean ± SD	2.0 ± 0.3	2.2 ± 0.2	t = 2.64	0.08	No statistically significant difference in central placental thickness between groups
Number of cotyledons, mean ± SD	13.1 ± 1.4	15.0 ± 1.2	t = 5.61	< 0.001	Reduced lobulation and villous branching in the placenta of mothers with MetS
Umbilical cord length, cm, mean ± SD	38.5 ± 5.8	46.4 ± 6.1	t = 5.48	< 0.001	Significantly shorter cords in pregnancies complicated by MetS
Umbilical cord diameter, mm, mean ± SD	11.2 ± 1.5	12.1 ± 1.3	t = 2.37	0.03	Significantly reduced diameter in MetS
Placental shape types^†^,^ m^edian (IQR)	1.0 (0.8–1.2)	1.2 (1.1–1.3)	U = 62	0.06	No statistically significant difference in placental shape distribution amongst groups

Placentae obtained from mothers with metabolic syndrome were consistently lighter and smaller in all major gross dimensions compared with those from healthy controls. The mean placental weight in the MetS group was 440 ± 52 g, whereas the mean placental weight in the control group was 515 ± 48 g, and this difference was statistically significant (p < 0.001). Placental length and breadth were also significantly reduced in the MetS group. The mean placental length was 17.4 ± 1.5 cm in the MetS group compared to 19.2 ± 1.6 cm in controls (p = 0.0002). Similarly, the mean placental breadth was 15.9 ± 1.3 cm in the MetS group vs. 17.5 ± 1.4 cm in the control group (p = 0.02). Placental thickness showed a mild reduction in the MetS group (2.0 ± 0.3 cm) compared with the control group (2.2 ± 0.2 cm); however, this difference did not reach statistical significance (p = 0.08).

Cotyledon count

The number of cotyledons was significantly lower in placentae from mothers with metabolic syndrome. The mean cotyledon count in the MetS group was 13.1 ± 1.4, whereas the control group showed a higher mean value of 15.0 ± 1.2, and this difference was statistically significant (p < 0.001).

Neonatal outcomes

Neonatal birth weight and the placenta-birth weight ratio are summarized in Table 3. Neonates born to mothers with metabolic syndrome had significantly lower mean birth weight (2.65 ± 0.34 kg) compared with those in the control group (3.05 ± 0.28 kg; p < 0.001). The placenta-birth weight ratio was also significantly lower in the MetS group (0.162 ± 0.02) compared with the control group (0.171 ± 0.03; p = 0.045).

**Table 3:** Comparison of neonatal outcomes between MetS and control groups The MetS group comprised 30 participants, and the control group comprised 30 participants. Statistical analysis was performed using the Independent samples t-test. A p-value < 0.05 was considered statistically significant MetS: metabolic syndrome; SD: standard deviation

Parameter	MetS group (n = 30)	Control group (n = 30)	Test statistic	P-value	Interpretation
Birth weight, kg, mean ± SD	2.65 ± 0.34	3.05 ± 0.28	t = 5.01	< 0.001	Significantly lower birth weight in neonates of MetS mothers
Placenta-birth weight ratio, mean ± SD	0.162 ± 0.02	0.171 ± 0.03	t = 2.03	0.045	Reduced placental efficiency in MetS pregnancies

Maternal surface hemorrhage

**Figure 2:**
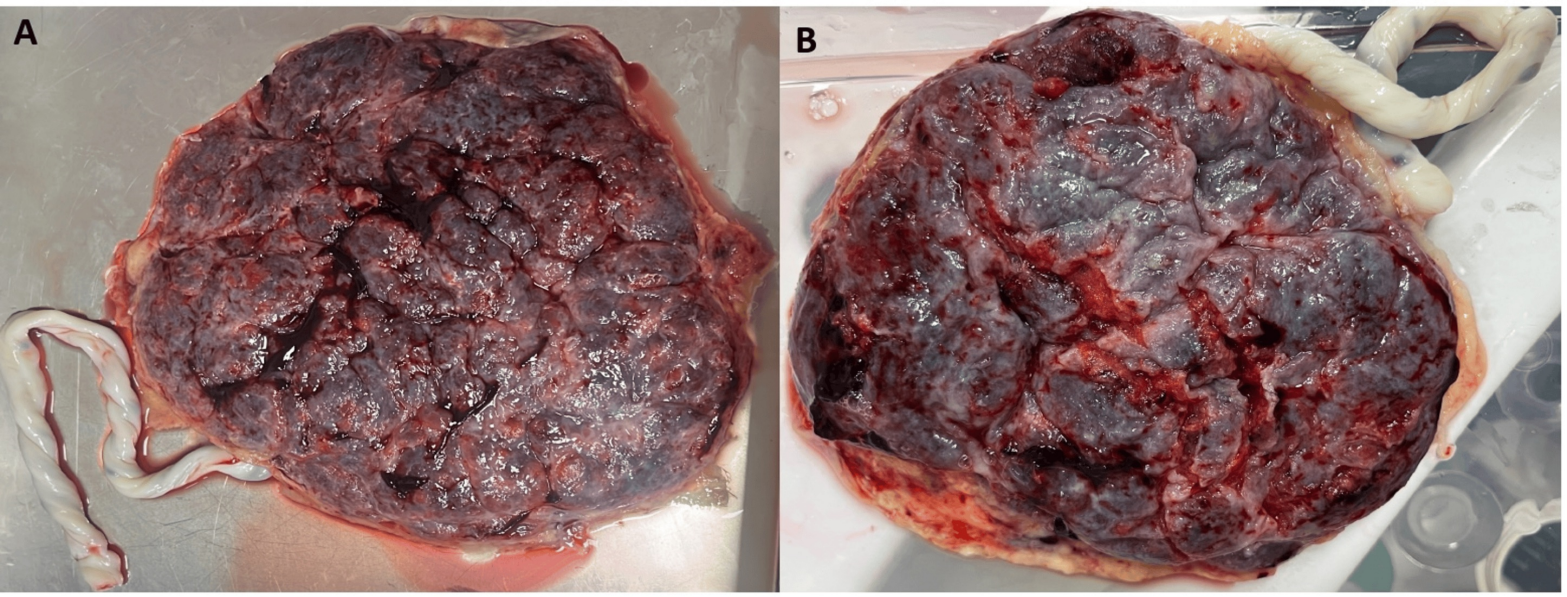
Comparison of maternal surface hemorrhage between MetS and control placentae Gross maternal surface of placenta from a mother with metabolic syndrome (A) showing extensive surface hemorrhage, dark congested areas, and marked red blood cell extravasation, indicating chronic placental hypoperfusion and vascular compromise. In contrast, the maternal surface of the control placenta (B) exhibits a relatively uniform appearance with minimal hemorrhage and preserved lobular architecture, consistent with normal placental circulation MetS: metabolic syndrome

Placentae from the MetS group more frequently demonstrated maternal surface hemorrhage and congestion compared with controls, as illustrated in Figure 2. Placentae from MetS pregnancies showed areas of surface hemorrhage and congestion, whereas placentae from the control group exhibited a relatively uniform maternal surface appearance.

Umbilical cord characteristics

**Figure 3:**
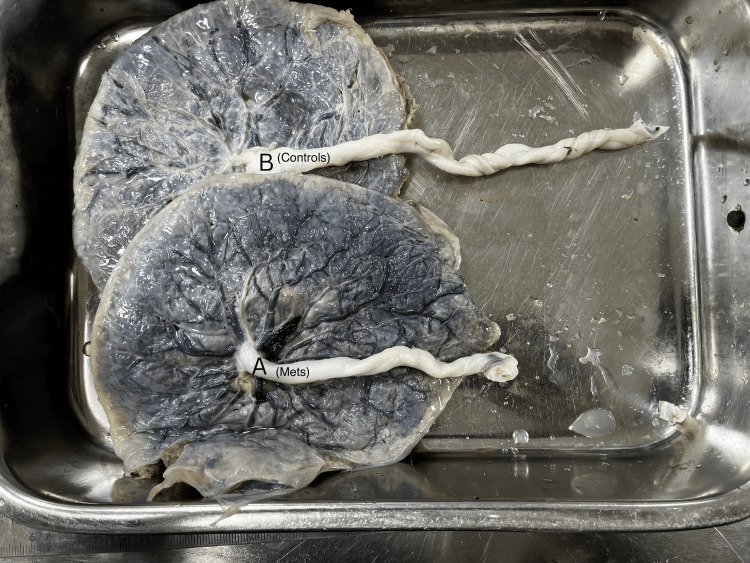
Gross appearance of umbilical cord length in MetS and control placentae Gross comparison of umbilical cord morphology in placentae from pregnancies complicated by metabolic syndrome (A) and from healthy control pregnancies (B). The placenta from the MetS group demonstrates a visibly shorter umbilical cord compared with the longer umbilical cord observed in the control placenta. Quantitative measurements of umbilical cord length are presented in Table 1 MetS: metabolic syndrome

Gross differences in umbilical cord morphology between the MetS and control groups are illustrated in Figure 3. Umbilical cord length was significantly shorter in pregnancies complicated by metabolic syndrome compared with healthy pregnancies; however, the mean cord lengths in both groups remained within the generally accepted normal range for term pregnancies. The mean umbilical cord length in the MetS group was 38.5 ± 5.8 cm compared to 46.4 ± 6.1 cm in the control group, and this difference was statistically significant (p < 0.001). The mean umbilical cord diameter was also significantly lower in the MetS group (11.2 ± 1.5 mm) compared with the control group (12.1 ± 1.3 mm; p = 0.03).

## Discussion

The fetus, placenta, and mother function as a closely integrated physiological unit, and alterations in any component can significantly influence fetal growth and pregnancy outcome. In the present study, pregnancies complicated by maternal MetS showed reductions in placental size, cotyledon number, and umbilical cord length, as well as lower neonatal birth weight and a reduced placenta-birth weight ratio. Collectively, these findings suggest compromised fetoplacental growth and efficiency in MetS pregnancies. Similar associations between placental morphology and fetal growth have been previously reported, underscoring the essential role of placental development and function in maintaining intrauterine homeostasis [1,2].

Placental growth and morphology are known to reflect the intrauterine metabolic and vascular environment. Reduced placental weight, as observed in the MetS group in this study, has been described in association with placental insufficiency and impaired fetal growth in several clinical and pathological investigations [3]. However, findings in the literature are heterogeneous. While some studies, particularly those focusing on isolated maternal diabetes or obesity, have reported increased placental weight attributed to compensatory hyperplasia or villous edema, others have demonstrated reduced placental mass in the presence of combined metabolic and vascular dysfunction [3-5]. These discrepancies likely reflect differences in metabolic phenotype, severity and duration of metabolic derangement, degree of metabolic control, and population characteristics. This study, focusing on the composite MetS phenotype, suggests that chronic metabolic and vascular stress may limit placental adaptive growth, resulting in reduced placental size rather than compensatory enlargement.

The observed reductions in placental length and breadth in the MetS group further support evidence of altered placental development. Placental surface dimensions are closely related to chorionic plate expansion and villous arborization, which together determine the functional exchange capacity of the placenta [9]. Previous morphometric studies have shown that reduced placental surface dimensions are associated with lower neonatal birth weight and diminished placental efficiency [10,11]. Although placental surface area was not directly measured in the present study, the consistent reduction in both length and breadth suggests restricted placental expansion in MetS pregnancies.

Beyond overall placental size, structural organization plays a critical role in placental function. The reduced cotyledon number observed in placentas from mothers with metabolic syndrome indicates altered placental subdivision. Cotyledons represent functional exchange units, and their number reflects gross placental lobulation and vascular organization [12]. While cotyledon count is an indirect marker and does not directly assess villous microarchitecture, similar reductions in placental subdivision and vascular development have been reported in pregnancies complicated by metabolic and hypertensive disorders [13-15]. Histopathological studies have further demonstrated impaired villous maturation and angiogenesis in placental insufficiency, providing a plausible mechanistic basis for the gross morphometric changes observed in this study.

Umbilical cord characteristics also differed significantly between the study groups. Umbilical cord length was significantly shorter in the MetS group compared with controls, although mean values in both groups remained within the generally accepted normal range for term pregnancies. Shorter umbilical cords have been associated with reduced fetal movement, chronic intrauterine stress, and adverse perinatal outcomes [16]. Large cohort and pathological studies have demonstrated associations between abnormal cord length and placental insufficiency, fetal growth restriction, and perinatal morbidity [17,18]. The reduced external umbilical cord diameter observed in the MetS group may further reflect compromised fetoplacental vascular development, although intravascular measurements were outside the scope of the present study.

Neonates born to mothers with metabolic syndrome in this study had significantly lower birth weight, accompanied by a reduced placenta-birth weight ratio, suggesting diminished placental efficiency. Similar findings have been reported in studies evaluating placental pathology in maternal metabolic disorders, including diabetes mellitus and hypertensive disease, where placental structural alterations have been linked to impaired nutrient transfer and fetal growth restriction [19,20]. Taken together, these findings reinforce the concept that maternal metabolic dysregulation adversely affects placental growth and function, with downstream consequences for fetal development.

Limitations

The present study has certain limitations that should be acknowledged. As a single-center study conducted at a tertiary care institution, the findings may not be fully generalizable to broader populations. Although the sample size was sufficient to detect differences in gross placental parameters, it was relatively modest and limited the ability to perform subgroup analyses based on individual components or severity of metabolic syndrome. Furthermore, the assessment was confined to gross placental morphology; detailed histopathological, stereological, or molecular analyses were not performed and would be necessary to confirm underlying microstructural and vascular changes. The cross-sectional design also precludes assessment of causal relationships or long-term maternal and neonatal outcomes.

Despite these limitations, the study was conducted and reported in accordance with the STROBE guidelines, ensuring transparent reporting of study design, methodology, and outcomes, and enhancing the interpretability and reproducibility of the findings [21].

## Conclusions

Maternal MetS is associated with restricted placental growth and impaired function, reflected by smaller placentae, fewer cotyledons, shorter umbilical cords, and reduced placental efficiency. These changes are associated with lower birth weight and an altered placenta-birth weight ratio, indicating compromised fetoplacental support. With the incidence of MetS rising among pregnant women, simple placental assessment, often the only tool available in low-resource labor rooms, can provide early clues to reduced placental reserve and fetal risk. Improving maternal metabolic health may therefore help to enhance neonatal outcomes and reduce the long-term intergenerational burden of cardiometabolic disease.
